# Neonatal Ross Procedure for Infective Endocarditis in a Dysplastic Aortic Valve After Pulmonary Artery Banding

**DOI:** 10.1016/j.jaccas.2025.106166

**Published:** 2025-11-28

**Authors:** Valentina Orioli, Lucio Careddu, Marianna Berardi, Valeria Francesca Mangerini, Francesco Dimitri Petridis, Gabriele Egidy Assenza, Marta Agulli, Andrea Donti, Gaetano Domenico Gargiulo, Emanuela Angeli

**Affiliations:** a Pediatric and Adult Congenital Heart Disease Cardiothoracic Surgery, Department of Cardio-thoracic and Vascular Medicine, IRCCS Azienda Ospedaliero-Universitaria di Bologna, Bologna, Italy; b Pediatric Cardiology and Adult Congenital Heart Disease Program, Department of Cardio-Thoracic and Vascular Medicine, IRCCS Azienda Ospedaliero-Universitaria di Bologna, Bologna, Italy; c Anaesthesiology and Intensive Care Unit, Department of Cardio-Thoracic and Vascular Medicine, IRCCS Azienda Ospedaliero-Universitaria di Bologna, Bologna, Italy; d Department of Medical and Surgical Sciences, DIMEC, University of Bologna, Bologna, Italy

**Keywords:** aortic coarctation, bicuspid aortic valve, cardiac risk, congenital heart defect, endocarditis, pediatric surgery, stenosis, ventricular septal defect

## Abstract

**Background:**

Neonatal Ross procedure is rarely performed, especially in the setting of active infective endocarditis and prior pulmonary artery banding.

**Case Summary:**

We present a rare case of a 2.5-month-old boy with a history of complex congenital heart disease, including aortic coarctation, severe aortic stenosis, and a ventricular septal defect, who developed infective endocarditis on a dysplastic bicuspid aortic valve.

**Discussion:**

This case highlights surgical decision-making and technical challenges in harvesting a pulmonary autograft after previous pulmonary artery banding in the presence of active infection.

**Take-Home Messages:**

Ross procedure can be feasible in neonates with complex anatomy and infection. Prior pulmonary artery banding increases technical complexity. Autograft resistance to reinfection supports its use in pediatric infective endocarditis.

## Visual Summary

**Figure:**
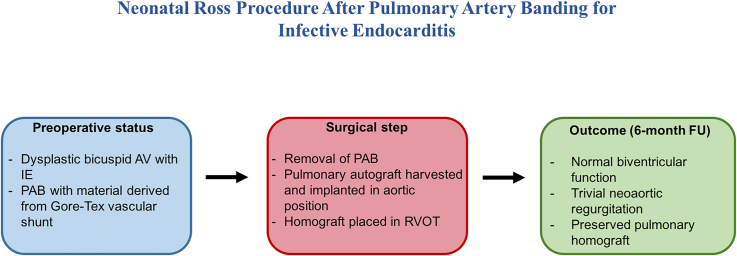

**Visual Summary:**
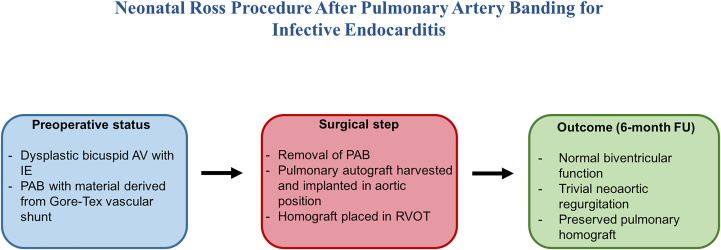
Schematic Overview of the Case: A Neonatal Ross Procedure was Successfully Performed After Previous Pulmonary Artery Banding The use of a Gore-Tex material for the PAB allowed safe removal without damaging the pulmonary trunk, enabling autograft harvest and implantation as a competent neoaortic valve. AV = atrioventricular; FU = follow-up; IE = infective endocarditis; PAB = pulmonary artery banding; RVOT = right ventricular outflow tract.

## History of Presentation

A male infant was born at 36 + 4 weeks’ gestation after in vitro fertilization. Prenatal imaging identified a complex congenital heart defect, including a large ventricular septal defect (VSD), left ventricular outflow tract obstruction, and cerebral ventriculomegaly. Limb anomalies such as polydactyly and syndactyly led to a diagnosis of Greig cephalopolysyndactyly syndrome (GLI3 mutation), confirmed by genetic analysis.

On day 2 of life, the patient developed progressive cyanosis and desaturation. Echocardiography revealed a severely dysplastic and stenotic bicuspid aortic valve with an aortic annulus of 6.5 mm (approximately 78% of normal for body surface area 0.23 m^2^, Z-score: −2), a 5-mm muscular apical VSD with left-to-right shunt, and critical coarctation of the aorta. Prostaglandin E1 infusion was initiated to maintain ductal patency.

Initial cardiac management included percutaneous balloon aortic valvuloplasty on day 30 of life, using 5 × 20 mm and 6 × 20 mm Tyshak Mini balloons (B. Braun Interventional Systems, Inc) under rapid pacing. This procedure resulted in a moderate degree of aortic regurgitation and a significant reduction in the transvalvular gradient (from 41 to 23 mm Hg).

Subsequently, on day 33 of life, the patient underwent surgical extended end-to-end repair of the aortic coarctation and pulmonary artery banding (PAB) in accordance with the Trusler rule. PAB was performed using material derived from a Gore-Tex vascular graft (W.L. Gore & Associates), generating a transpulmonary gradient of 55 mm Hg. Post-PAB echocardiography confirmed normal biventricular function and good branch perfusion.

Despite initial stabilization, a follow-up echocardiogram revealed a recurrence of significant isthmic stenosis. On October 2, 2024, percutaneous balloon angioplasty of the aortic isthmus was performed using a 6 × 20 mm Tyshak Mini balloon. The postprocedural angiogram showed improved caliber.

## Past Medical History

In addition to complex congenital heart disease, the patient experienced significant extracardiac comorbidities. On day 2 of life, he developed necrotizing enterocolitis requiring urgent laparotomy with resection of the affected ileum, creation of double ileostomy, and appendectomy.

At 2 months of age, a ventriculoatrial (VA) shunt was placed for progressive hydrocephalus associated with cerebral ventriculomegaly.

At 5 months of age, the patient developed persistent fever. Initially managed by the primary care physician with symptomatic treatment and antibiotics, his symptoms persisted over several days. Multiple outpatient evaluations were conducted, including febrile episodes from December 26, 2024, with a pediatric emergency department visit on December 28, 2024, revealing no positive findings on rapid viral testing or urinalysis. Despite oral amoxicillin therapy, fevers recurred every 6 to 8 hours.

On January 2, 2025, during a routine cardiology follow-up, transthoracic echocardiography unexpectedly revealed a pedunculated, echogenic mass on the aortic valve, raising suspicion for endocarditis. Given the clinical history and imaging findings, the patient was admitted for further evaluation and surgical planning.

## Investigations

On admission, a comprehensive transthoracic echocardiogram showed the following findings.

**Figure 1:**
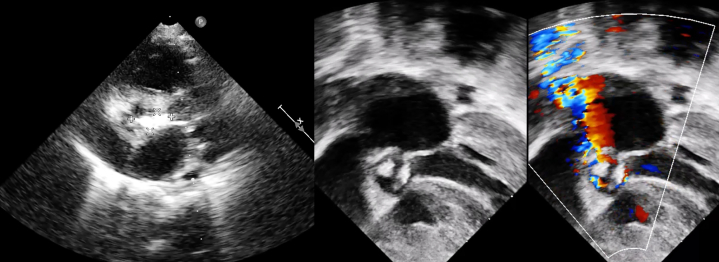
Preoperative Transthoracic Echocardiogram, Parasternal Long Axis View, Showing Aortic Valve Endocarditis
• Severe aortic stenosis with peak/mean gradients of 88/42 mm Hg; absence of subaortic stenosis.
• PAB in place with a peak gradient of 62 mm Hg, but preserved pulmonary trunk morphology without distorsion or torsion.
• Competent pulmonary valve; no regurgitation.
• No significant recoarctation.
• Accelerated flow at the aortic isthmus with a peak Doppler gradient of 28 mm Hg.
• Visualized tip of the VA shunt in the right atrium.

• Restrictive subaortic and apical VSDs, each measuring <3 mm, with exclusive left-to-right shunt.
• Moderate biventricular hypertrophy with preserved systolic function; left ventricular ejection fraction was 63%.
• Dysplastic, thickened bicuspid aortic valve with a pedunculated, oval-shaped, hyperechogenic mass attached to the cusps, prolapsing in diastole; the vegetation measured 1.5 × 1.0 cm (Figure 1).

Blood cultures were performed and returned positive for *Enterococcus faecalis*. The patient was started on targeted intravenous antibiotic therapy in coordination with infectious disease specialists. Throughout the course of infective endocarditis, the patient remained neurologically stable, with no evidence of cerebral infarction.

## Management

**Figure 2:**
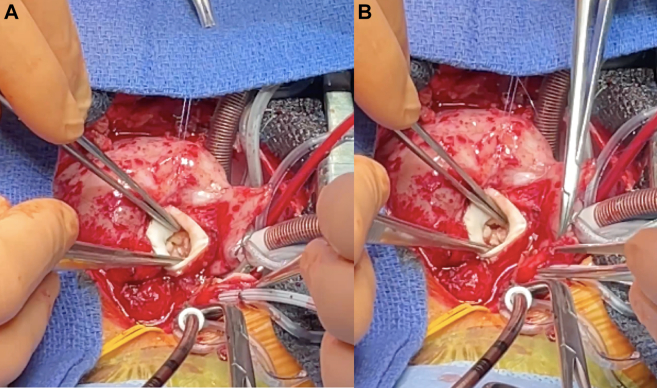
Intraoperative Findings (A) Aortic valve with dysplastic cusps and vegetations. (B) Bicuspid morphology of the valve with fissured and infected leaflets. Cephalocaudal orientation is maintained.

**Figure 3:**
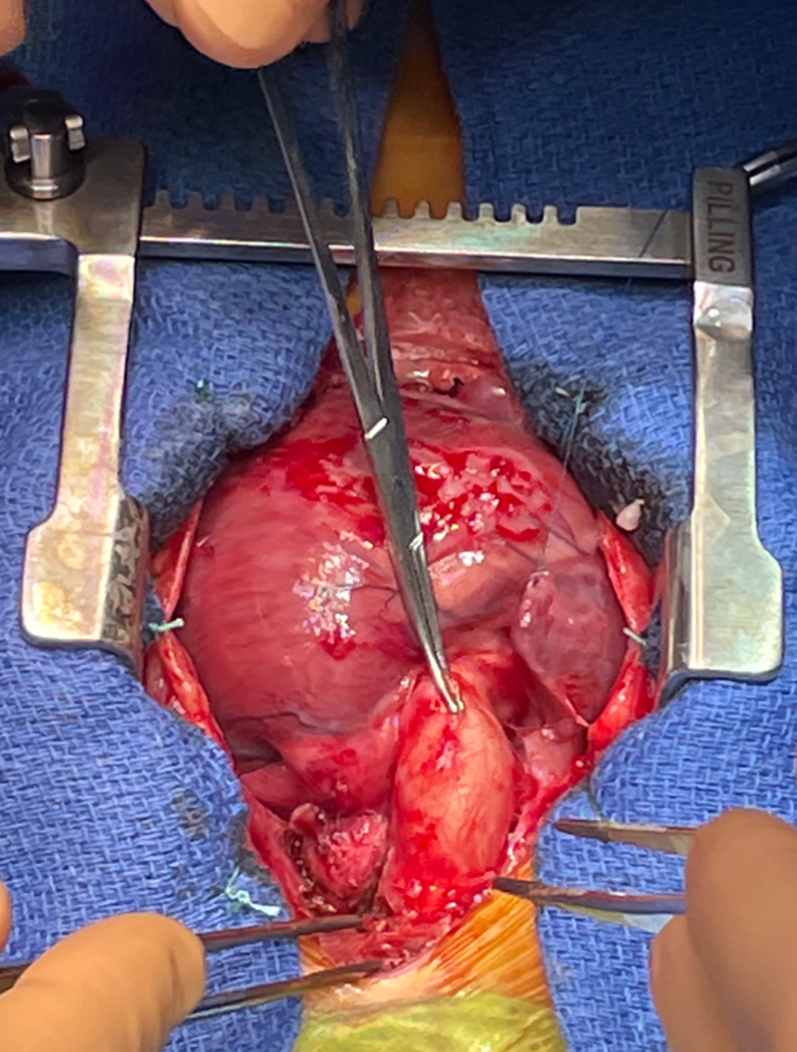
Intraoperative Image of the Pulmonary Artery Banding Before Removal The band consisted of a Gore-Tex graft, which was atraumatically dissected, preserving pulmonary trunk morphology.

Given the presence of infective endocarditis on a severely dysplastic aortic valve, a stable vegetation despite medical therapy, and progressive obstruction, surgical intervention was indicated (Figure 2). The presence of prior PAB posed a significant technical challenge for a Ross procedure, which requires harvesting the native pulmonary valve as an autograft (Figure 3). Alternative surgical strategies, including aortic valvuloplasty with or without a Konno procedure, were considered. The multidisciplinary team ultimately elected to proceed with the Ross operation, given the imperative to eradicate infection and the advantage of avoiding prosthetic material in a growing child. An intraoperative evaluation of the VSD was planned to determine the necessity for closure.

## Ross Procedure

On January 30, 2025, a median sternotomy was performed (Video 1). Cardiopulmonary bypass was established between the ascending aorta and the caval veins, with an oxygenator and systemic hypothermia at 25 °C. The ascending aorta and pulmonary trunk were fully mobilized. After aortic cross-clamping, aortotomy was carried out and cold crystalloid cardioplegia was infused through the coronary ostia. The ascending aorta was transected 2 cm above the sinotubular junction. Two coronary buttons containing the ostia were excised en bloc. The PAB was removed. Because the PAB had been constructed using material derived from a Gore-Tex vascular graft, it could be dissected and removed without injuring or deforming the pulmonary trunk. This preserved the pulmonary autograft's integrity and valve competence, enabling its safe harvest. The pulmonary valve and trunk were excised. Hemostasis was achieved. The diseased aortic valve was removed entirely, with vegetations and fissured leaflets identified and sent for microbiological analysis. The pulmonary autograft was implanted in the aortic position with running 5-0 Prolene sutures. Coronary reimplantation was performed, left coronary artery was reimplanted with the Trap door technique, and reimplantation of the right coronary artery was performed with a Button technique. The distal anastomosis of the autograft to the ascending aorta was completed using 6-0 Prolene sutures. A 16-mm pulmonary homograft was then anastomosed proximally to the right ventricle and distally to the pulmonary bifurcation. After deairing, the aortic cross-clamp was removed and sinus rhythm resumed spontaneously. No residual gradient was noted between the left ventricle and aorta.

Intraoperative exploration revealed that the VSD was already closed by accessory tissue.

## Follow-Up

**Figure 4:**
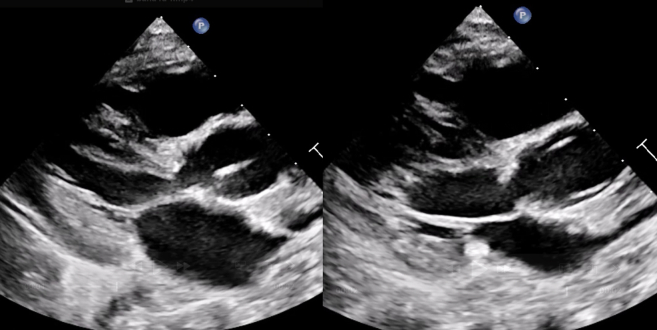
Postoperative Transthoracic Echocardiogram, Parasternal Long Axis View, Showing Newly Identified Subaortic Stenosis in Systole and Diastole

At 6 months postoperatively, the patient was clinically well with appropriate weight gain and feeding. Echocardiography showed the following: normal biventricular function; trivial neoaortic regurgitation; aortic annulus measuring 13 mm; subaortic membrane, newly identified, located 12 mm below the aortic annulus, with a 30-mm Hg gradient (Figure 4); competent pulmonary homograft with 30-mm Hg gradient; and no evidence of recoarctation.

Importantly, no neurologic complications occurred. Neurocognitive development was appropriate for age.

## Discussion

The Ross procedure is infrequently performed in the neonatal period, particularly in the setting of active infective endocarditis and previous PAB. This case demonstrates that a pulmonary autograft can be safely harvested even after PAB, provided the PAB is performed with material derived from a removable Gore-Tex vascular graft.

According to current guidelines, the Ross procedure is a class IIb recommendation in young patients with aortic valve disease when long-term anticoagulation is contraindicated or undesirable, and in selected cases of endocarditis where autologous tissue is preferred.^1^ In the pediatric population, this recommendation is extrapolated from expert consensus and limited series.

Reports of the Ross procedure in the setting of active infective endocarditis are rare, particularly in neonates. A few case series and case reports describe successful outcomes in older children and adolescents with endocarditis.^2^, ^3^, ^4^ Al-Baradai et al^5^ reported a case of neonatal infective endocarditis due to *Streptococcus* species requiring urgent Ross operation with favorable midterm follow-up.

El-Hamamsy et al^6^ emphasized the resistance of the pulmonary autograft to reinfection in adults undergoing the Ross procedure for endocarditis, a feature potentially valuable in pediatric patients.

The challenge was further compounded by the presence of a prior pulmonary artery band. To date, there is extremely limited literature describing Ross procedures performed after prior PAB, particularly in the neonatal population. The removal of the band and safe harvest of a structurally preserved pulmonary autograft require careful dissection and may pose risks of distortion or injury to the autograft.^7^^,^^8^

The decision to perform a Ross procedure in this setting was driven by the need to eradicate infection, provide durable relief of outflow tract obstruction, and avoid prosthetic material in a growing child.^9^^,^^10^

**Take-Home Messages**

• Ross procedure can be feasible in neonates with complex anatomy and infection.
• Prior pulmonary artery banding increases technical complexity. Autograft resistance to reinfection supports its use in pediatric infective endocarditis.

## Funding Support and Author Disclosures

The authors have reported that they have no relationships relevant to the contents of this paper to disclose.

## Appendix

Video 1 Ross procedure
